# Analgesia nociception index is an indicator of laparoscopic trocar insertion-induced transient nociceptive stimuli

**DOI:** 10.1515/med-2024-0933

**Published:** 2024-04-25

**Authors:** Jun Liu, Zhuodan Wang, Wan Huang, Nan Cheng, Weiqiang Chen, Weijun Wu, Shangrong Li

**Affiliations:** Department of Anesthesiology, The Third Affiliated Hospital, Sun Yat-sen University, Guangdong Province, 510630, China; Department of Anesthesiology, The Second Affiliated Hospital, Guangzhou Medical University, Guangzhou City, Guangdong Province, 510260, China; Department of Anesthesiology, Sun Yat-sen University Cancer Center, Guangzhou City, Guangdong Province, 510060, China; Shenzhen Mindray Bio-Medical Electronics Co., Ltd., Shenzhen City, Guangdong Province, 518000, China; Department of Anesthesiology, The Third Affiliated Hospital, Sun Yat-sen University, 600 Tianhe Road, Guangzhou City, Guangdong Province, 510630, China

**Keywords:** pain monitoring, analgesia nociception index, general anesthesia, target-controlled infusion, remifentanil

## Abstract

**Objective:**

This study aimed to investigate whether analgesia nociception index (ANI) could be an indicator of perioperative pain during laparoscopic trocar insertion.

**Methods:**

A total of 280 participants of anesthesia receiving laparoscopic surgery were enrolled. Anesthesia induction and maintenance were performed using the Marsh model for target propofol and the Minto model for remifentanil. Systolic blood pressure (SBP), heart rate (HR), and ANI were recorded at skin incision, the first-, second, the last-trocar insertion, and 5 min after the last trocar insertion.

**Results:**

ANI was significantly different among the five groups in the last four time points (all *P* < 0.05). Pearson’s correlation showed that ANI was negatively correlated with SBP (*r* = −0.114, *P* = 0.077) and HR (*r* = −0.247, *P* < 0.001). The area under the curve of ANI was positively correlated with those of SBP (*r* = 0.493, *P* < 0.001) and HR (*r* = −0.420, *P* < 0.001). Multivariate logistic regression showed that the ANI was an independent factor associated with intraoperative hemodynamic adverse events only at 5 min after the last trocar insertion.

**Conclusions:**

Under general anesthesia, the change in ANI was consistent with changes in the balance between analgesia and nociceptive stimuli. The ANI can reflect the extent of transient pain but had a poor predictive performance for hemodynamic adverse events.

## 1 Introduction

Pain is considered the fifth vital sign by the American Pain Society [1]. Previous studies have reported that about 80% of the surgical patients suffer from various pain-related problems and complications due to inappropriate pain management [2–4]. The incidence of perioperative acute pain is up to 30–50% [5,6].

In general anesthesia, analgesic drugs can inhibit nociceptive stimuli-induced excitation of sympathetic tone, thereby achieving a nociception–anti-nociception balance [7]. An excessive or insufficient dose of sedative or analgesic drugs can lead to a series of adverse reactions, affecting the postoperative recovery time, hospitalization time, and prognosis. Therefore, the optimal strategy is to use a minimum effective dosage of the analgesic drug to maintain the appropriate anesthesia depth [8]. Analgesia monitoring is an important component of clinical anesthesia monitoring [9]. Accurate analgesia monitoring can help optimize the use of analgesics.

Clinically, the bispectral (BIS) index has been used in the assessment of sedation level [10,11]. Several monitoring tools for analgesia assessment in anesthetized patients have been developed [9], such as Surgical Pleth Index [12] and skin conductance [13]. However, due to the complexity of the pain mechanism and the anesthesia state of the patient, there is still no well-recognized indicator for monitoring perioperative pain [14].

In 2011, Logier proposed the concept of analgesia nociception index (ANI) based on the theory of heart rate variability (HRV) [15]. ANI is a non-invasive tool developed for nociception assessment [16]. The ANI is a 0–100 index measuring the activity of the parasympathetic nervous system based on the HRV, which can timely reflect the analgesia and nociceptive stimuli during general anesthesia [16,17]. The ANI monitor continuously records the electrocardiographic signal and quantitatively assesses the respiratory HRV [18]. It has been shown that the changes in ANI can reflect the levels of the nociceptive stimulus [17]. In addition, compared to systolic blood pressure (SBP) and heart rate (HR), ANI is more sensitive to moderate nociceptive stimuli in propofol-anesthetized patients [19]. Nevertheless, a study also reports that ANI cannot distinguish the degree of acute pain [20]. Compared to the invasive hemodynamic changes, the predictive value of ANI is not prominent [21]. A randomized clinical trial demonstrates that intraoperative analgesia guided by ANI monitoring does not reduce postoperative pain after laparoscopic cholecystectomy [22]. These results suggest that whether the ANI can be used as an indicator for nociceptive stimuli remains to be further investigated. We hypothesize that ANI may be an indicator for perioperative pain during laparoscopic trocar insertion. Therefore, this study aimed to evaluate the effect of ANI on transient pain monitoring during the perioperative period.

## 2 Methods

### 2.1 Study design and participants

This was a prospective, multicenter, randomized, single-blind, controlled trial, and was registered in the West China Hospital of Sichuan University, Chengdu, China (http://www.chictr.org.cn/; registration number: ChiCTR-RDD-17011172). This multicenter trial was performed in the Third Affiliated Hospital of Sun Yat-sen University, the Affiliated Tumor Hospital of Sun Yat-sen University, and the Second Affiliated Hospital of Guangzhou Medical University.

The inclusion criteria were (1) patients undergoing elective abdominal laparoscopic surgery (at least three trocars insertion) with tracheal intubation under general anesthesia, (2) patients with the American Society of Anesthesiologists (ASA) Patient Physical Status Classification of ASA I or ASA II, and (3) hospitalized patients between the ages of 18 and 60. Exclusion criteria were (1) without the capacity to make a juridical act; (2) preoperative visceral dysfunction; (3) long-term use of hormonal, psychoactive, or opioid drugs; (4) arrhythmia affecting the P–P interval (non-sinus heart rhythm or with a pacemaker, first degree or higher atrioventricular block, atrial fibrillation); (5) regular use of β-blockers; (6) history of alcohol abuse; and (7) hypertension (pre-operative irregular medication or regular medication, but SBP still higher than 160 mmHg 3 days before surgery).

**Figure 1:**
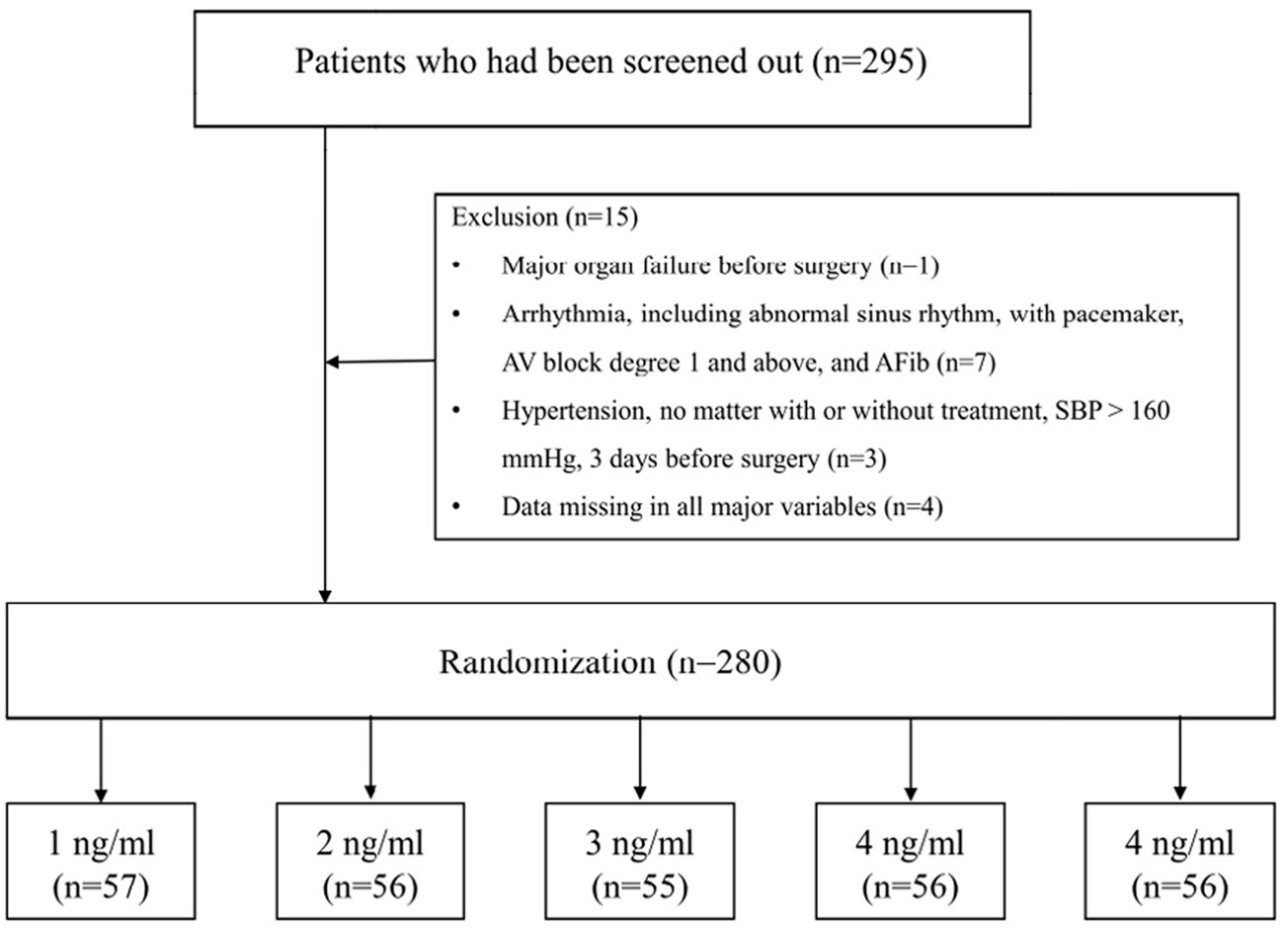
Flow chart of patient enrollment.

Randomization was carried out using a random number table, and the participants were blinded to the group assignments. A total of 295 patients receiving laparoscopic surgery were recruited from the three hospitals between May 2017 and February 2018. According to the criteria, 15 patients were excluded. Finally, 280 eligible participants were enrolled and randomized into five groups receiving different target-controlled infusion (TCI) concentrations of remifentanil [23,24]: 1 ng/mL (*n* = 57), 2 ng/mL (*n* = 56), 3 ng/mL (*n* = 55), 4 ng/mL (*n* = 56), and 5 ng/mL (*n* = 56) (Figure 1).

### 2.2 Sample size estimation

The primary objective of this study was to investigate the correlations between the ANI and immediate pain intensity of TCI concentration of remifentanil at the start of trocar insertion, and 5 min after the last trocar insertion. According to the preliminary results of a previous study [25], a necessary sample size of 220 was estimated to detect a correlation between the intraoperative nociceptive reflexes and the intensity of immediate pain with a power of 0.8. Therefore, the sample size of each group was estimated to be 44 by using a different sample size formula. Considering the 20% dropout rate during clinical trials, the final sample size of each group was determined to be 55 cases.

Correlations were calculated as Spearman correlations.

### 2.3 Study procedure

All patients did not receive preoperative sedatives and anesthetics. The blood pressure, electrocardiogram, oxyhemoglobin saturation by pulse oximetry, BIS (T5 Patient Monitor, BeneView T5 Patient Monitor; Mindray, China), and ANI were monitored after admission to the operating room.

The patient was given oxygen (5 L/min) by face mask, and Ringer’s solution (10 mL/kg) was intravenously administered via the upper extremity venous access. Anesthesia was induced with intravenous midazolam (0.05 mg/kg), and intravenous TCI of propofol and remifentanil using the Marsh [26] and the Minto [27] pharmacokinetic parameters. After the patients lose consciousness, cisatracurium (0.2–0.6 mg/kg) was given intravenously, and the tracheal intubation was accomplished after full muscle relaxation. Mechanical ventilation was performed with the following setting: volume-controlled ventilation mode, tidal volume = 8–10 mL/kg, respiratory rate = 12 bpm, and the respiratory ratio = 1:2. The P_ET_CO_2_ was maintained at 35–45 mmHg during the operation. After tracheal intubation was completed, an arterial catheter was inserted into the radial artery for invasive arterial pressure monitoring. Then the patient was supine for 5 min, the infusion of remifentanil was stopped and the propofol was injected to maintain the depth of anesthesia, with the BIS of 40–60.

After disinfection by the surgeon, the anesthesiologist performed TCI of remifentanil at different concentrations. During the course of the study (from skin incision to 5 min after the last trocar insertion), anesthesia was maintained only by intravenous infusion. After that, anesthesia was performed according to the needs in the surgical procedure.

### 2.4 Data collection

**Figure 2:**
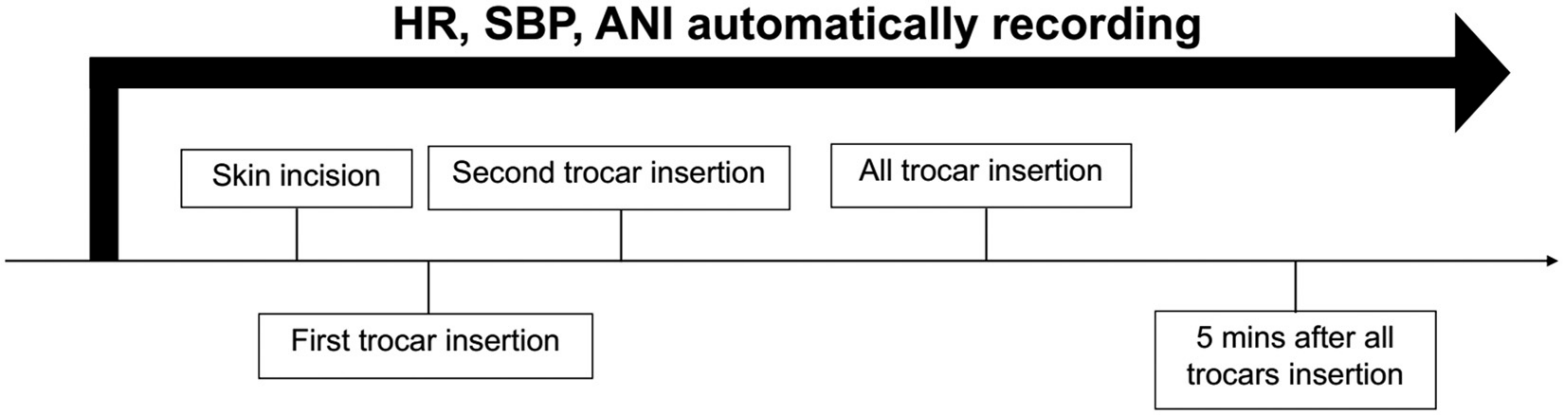
Timeline of HR, SBP, and ANI recording during laparoscopic trocar insertion.

The intraoperative data, including HR, SBP, and ANI, were automatically recorded by the instruments at five time points (skin incision, first trocar insertion, second trocar insertion, the last trocar insertion, and 5 min after the last trocar insertion, Figure 2). The curves of SBP, HR, and ANI were plotted from skin incision to 5 min after the last trocar insertion by the instruments, and the area under the curve (AUC) was analyzed.

### 2.5 Hemodynamic adverse events

Hemodynamic adverse events (hypertension, hypotension, tachycardia, bradycardia) were recorded during surgery [28]. The mean value of non-invasive SBP and HR measured within 1 min before the skin incision was defined as the baseline value.

The hemodynamic adverse events were treated according to the normalization process as follows [4,29]:

1) Transient hemodynamic adverse events, such as hypertension (SBP >160 mmHg), tachycardia (HR >100 bpm), hypotension (SBP <90 mmHg), or bradycardia (HR <50 bpm), occurring during the study would not be treated. If the hemodynamic adverse events lasted for more than 5 min, the concentrations of propofol would be adjusted to maintain BIS at 40–60, with an adjustment range of 0.5 μg/mL. The concentration of remifentanil was adjusted according to the hemodynamic instability with an adjustment range of 1 ng/mL. If hemodynamic is still uncontrolled after adequate sedation (BIS at 40–60) and adequate analgesia, the patient should be withdrawn from the study, and hypertension was treated with intravenous urapidil hydrochloride injection (10–15 mg, could be repeated after 5 min); tachycardia was treated with intravenous esmolol (20 mg); hypotension was treated with infusion of compound sodium lactate (200 mL) within 10 min, and intravenous injection of dopamine (2 mg) was also given if necessary; bradycardia was treated with intravenous injection of atropine (0.5 mg). The above treatments can be repeated if necessary. The ANI values measured within 30 min after injection of esmolol or atropine would be inaccurate and excluded for analysis.

### 2.6 Statistical analysis

Continuous variables were presented as mean ± standard deviation and were compared by one-way ANOVA. For the five TCI concentration groups of remifentanil, linear trend analysis was used to confirm the linear change among the groups. For the comparisons of mean values of two groups, Student’s independent *t*-test was used. Categorical data were presented as number and percentage (%), and were compared by the Chi-square test or Fisher’s exact test (if any expected value lower than 5 was observed). Pearson’s correlation and partial correlation coefficients were used to investigate the correlations among variables. Multivariate logistic regression was used to estimate the odds ratio (and its 95% CI) of independent variables to overall hemodynamic instability. Receiver operating characteristic curve (ROC) analysis was used to investigate the predictive effectiveness of continuous variables to dichotomous results, and the AUC was reported. A *P-*value lower than 0.05 would be recognized as statistical significance. All analyses were performed using IBM SPSS Version 20 (SPSS Statistics V20, IBM Corporation, Somers, NY, USA).


**Ethical approval**: This study was approved by the Third Affiliated Hospital of Sun Yat-sen University clinical medical research ethics committee.
**Informed consent:** Written informed consent was obtained from all participants.

## 3 Results

### 3.1 Patient’s characteristics

A total of 280 participants receiving laparoscopic surgery (mean age = 44.73 ± 10.05 years, BMI = 22.46 ± 3.05 kg/m^2^, the mean number of trocar insertions = 3.90 ± 0.88) were enrolled and randomly divided into five groups with different remifentanil concentrations (1, 2, 3, 4, 5 ng/mL). As shown in Table 1, there was no significant difference in patients’ characteristics, including, age, gender, height, weight, BMI, and the number of trocars among the five groups (all *P* > 0.05), indicating their comparability.

**Table 1:** Patients’ characteristics

Parameters	1 ng/mL (*n* = 57)	2 ng/mL (*n* = 56)	3 ng/mL (*n* = 55)	4 ng/mL (*n* = 56)	5 ng/mL (*n* = 56)	*P*
Age, years	44.02 ± 9.05	45.45 ± 10.43	43.71 ± 9.91	46.38 ± 9.63	44.07 ± 11.21	0.581
**Gender**						0.404
Male	18 (31.58)	19 (33.93)	23 (41.82)	15 (26.79)	23 (41.07)	
Female	39 (68.42)	37 (66.07)	32 (58.18)	41 (73.21)	33 (58.93)	
BMI, kg/m^2^	22.56 ± 2.86	22.27 ± 3.49	22.09 ± 2.81	22.35 ± 2.46	23.00 ± 3.49	0.564
Number of trocars	3.98 ± 0.88	3.76 ± 0.88	3.82 ± 0.86	3.98 ± 0.87	3.95 ± 0.94	0.582
**Organization**						1.000
A	18 (31.58)	19 (33.93)	19 (34.55)	18 (32.14)	20 (35.09)	
B	24 (42.11)	23 (41.07)	22 (40.00)	23 (41.07)	23 (40.35)	
C	15 (26.32)	14 (25.00)	14 (25.45)	15 (26.79)	14 (24.56)	

Organization means the three hospitals. All patients were from these three hospitals and allocated into five groups.

### 3.2 Safety of low concentration remifentanil

In this study, we designed the five remifentanil concentration groups (1–5 ng/mL) based on the TCI method as previously described [23,24]. To evaluate the safety of low concentration remifentanil of 1 and 2 ng/mL, a preliminary study was conducted. Another 32 patients (8 males and 24 females, mean age = 42.53 ± 11.39 years [range: 22–60]) were included and divided into 1 ng/mL group and 2 ng/mL group (*n* = 16 for each group). Their SBP and HR were monitored and recorded at five time points as described above. The results showed that only one patient had continued perioperative hypertension. After antihypertensive treatment, the vital signs returned to stable and the surgery was completed. The other four patients had transient hypertension, but their vital signs quickly stabilized and the surgery was not affected. These results suggested that low concentration remifentanil of 1 and 2 ng/mL was safe and effective for anesthesia during laparoscopic surgery.

### 3.3 Patient’s SBP, HR, and ANI at different time points

The intraoperative data, including SBP, HR, and ANI, were monitored at five time points. Significant differences were observed in SBP at the first and second trocar insertion, in HR and ANI at the first, second, last trocar insertion, and 5 min after the last trocar insertion (all *P* < 0.05, ANOVA, Tables 2 and 3). To further evaluate the changing trend, linear trend analysis was performed. As shown in Table 2, the linear trend was significant in all the variables except for SBP at skin incision and HR at 5 min after the last trocar insertion. The lower concentration of remifentanil was associated with higher SBP, HR, and lower ANI (all *P* < 0.05, linear trend analysis, Table 3).

**Table 2:** Patient’s SBP and HR index at different monitoring times

	1 ng/mL	2 ng/mL	3 ng/mL	4 ng/mL	5 ng/mL	*P*	
Parameters	(*n* = 57)	(*n* = 56)	(*n* = 55)	(*n* = 56)	(*n* = 56)	ANOVA	Linear trend
**SBP**							
Skin incision	117 ± 22	108 ± 18	112 ± 24	108 ± 19	107 ± 16	0.080	0.526
First trocar insertion	135 ± 26	127 ± 19	126 ± 22	121 ± 21	115 ± 19	<0.001	<0.001
Second trocar insertion	142 ± 25	137 ± 20	137 ± 21	131 ± 24	122 ± 21	<0.001	0.002
The last trocar insertion	144 ± 25	140 ± 24	141 ± 21	140 ± 25	130 ± 22	0.074	0.002
5 min after the last trocar insertion	139 ± 22	141 ± 25	142 ± 22	140 ± 25	136 ± 19	0.707	0.034
**HR**							
Skin incision	64 ± 10	59 ± 10	59 ± 11	59 ± 12	59 ± 11	0.125	0.020
First trocar insertion	68 ± 11	62 ± 11	60 ± 10	61 ± 13	62 ± 15	0.007	<0.001
Second trocar insertion	69 ± 12	63 ± 12	61 ± 11	61 ± 13	62 ± 12	0.015	<0.001
The last trocar insertion	69 ± 11	63 ± 13	61 ± 12	61 ± 11	62 ± 11	0.009	0.012
5 min after the last trocar insertion	67 ± 14	62 ± 10	61 ± 12	60 ± 11	61 ± 14	0.047	0.404

SBP, systolic blood pressure; HR, heart rate; ANI, analgesia-injury stimulus index.

**Table 3:** Patient’s ANI at different monitoring times

	1 ng/mL	2 ng/mL	3 ng/mL	4 ng/mL	5 ng/mL	*P*	
Parameters	(*n* = 57)	(*n* = 56)	(*n* = 55)	(*n* = 56)	(*n* = 56)	ANOVA	Linear trend
**ANI**							
Skin incision	67 ± 19	72 ± 21	75 ± 16	71 ± 21	71 ± 21	0.443	0.041
First trocar insertion	51 ± 20	54 ± 19	65 ± 20	67 ± 20	69 ± 22	<0.001	0.013
Second trocar insertion	51 ± 18	52 ± 17	60 ± 20	59 ± 17	60 ± 17	0.017	0.013
The last trocar insertion	50 ± 16	54 ± 21	59 ± 16	58 ± 17	60 ± 21	0.022	0.005
5 min after the last trocar insertion	60 ± 20	68 ± 19	72 ± 17	69 ± 19	68 ± 20	0.016	0.015

Correlation coefficient analysis showed that ANI was negatively correlated with SBP and HR, while SBP was positively correlated with HR (Table 4, the original index, including correlation and partial correlation analyses).

**Table 4:** Pearson’s correlation and partial correlation coefficients

	Original index (first trocar insertion)		AUC (from skin incision to 5 min after the last trocar insertion)	
Pairs	*r*	*P*	*r*	*P*
**Correlation**				
ANI–SBP	−0.114	0.077	0.493	<0.001
ANI–HR	−0.247	<0.001	0.420	<0.001
SBP–HR	0.135	0.037	0.669	<0.001
**Partial correlation**				
ANI–SBP	−0.083	0.201	0.315	<0.001
ANI–HR	−0.252	<0.001	0.137	0.035

SBP, systolic blood pressure; HR, heart rate; ANI, analgesia-injury stimulus index; AUC, area under the curve.

The AUC (from skin incision to 5 min after the last trocar insertion) was generated and compared among the three variables (ANI, HR, and SBP). There was no significant difference in the AUC of ANI among the five groups (*P* = 0.099). However, the AUC of SBP, HR, and ANI were all significantly positively correlated with each other (Table 4, AUC, including correlation and partial correlation analyses; all *P* < 0.05).

In further analysis of gender difference in ANI (Table A1), among 25 results (5 remifentanil concentrations crossed 5 time points), only 3 (12%) reached statistical significance. These results suggested that gender did not affect ANI.

### 3.4 Remifentanil concentration, ANI, and hemodynamic adverse events

The intraoperative hemodynamic adverse events are summarized in Table 5. There was no significant difference in the incidence of hemodynamic adverse events among the five groups (all *P* > 0.05, Table 5).

**Table 5:** Hemodynamic adverse events

Adverse events	1 ng/mL (*n* = 57)	2 ng/mL (*n* = 56)	3 ng/mL (*n* = 55)	4 ng/mL (*n* = 56)	5 ng/mL (*n* = 56)	*P*
Hypertension	1 (1.75)	3 (5.36)	2 (3.64)	1 (1.79)	1 (1.69)	0.734
Hypotension	3 (5.26)	1 (1.79)	1 (1.82)	1 (1.79)	3 (5.08)	0.646
Tachycardia	0	0	0	0	0	—
Bradycardia	1 (1.75)	1 (1.79)	1 (1.82)	0 (0.00)	0 (0.00)	0.533
Overall hemodynamic adverse events	4 (7.02)	5 (8.93)	4 (7.27)	2 (3.57)	4 (6.78)	0.829

Multivariate logistic regression showed that the ANI at 5 min after the last trocar insertion was associated with overall hemodynamic adverse events (OR: 1.03, 95% CI: 1.00–1.06; *P* = 0.045, Table 6).

**Table 6:** Multivariate logistic regression results of independent factor associated with overall hemodynamic adverse events

Parameters	Estimated OR (95% CI)	*P*
ANI, 5 min after the last trocar insertion	1.03 (1.00–1.06)	0.045
**Remifentanil group**		0.859
1 ng/mL	ref.	—
2 ng/mL	0.82 (0.18–3.63)	0.789
3 ng/mL	0.64 (0.14–2.88)	0.561
4 ng/mL	0.36 (0.06–2.20)	0.271
5 ng/mL	0.75 (0.17–3.30)	0.700
**Gender**		
Male	ref.	—
Female	2.08 (0.45–9.67)	0.352
Age	1.02 (0.97–1.08)	0.454
Height, cm	1.07 (0.97–1.19)	0.189
Weight, kg	1.01 (0.95–1.07)	0.728

Multivariate logistic regression adjusting for remifentanil concentration, age, gender, height, and weight; CI, confidence interval; OR, odds ratio.

**Figure 3:**
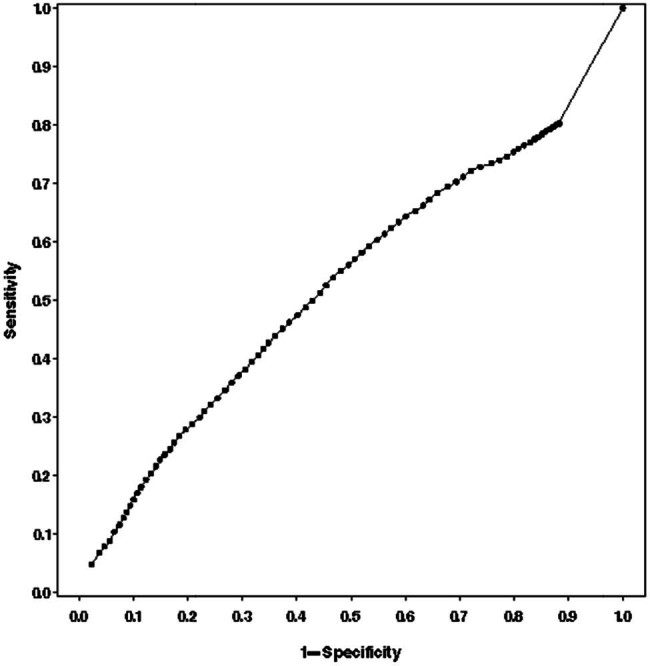
ROC analysis of the predictive performance of ANI for hemodynamic adverse events. AUC = 0.527 (95% CI: 0.522–0.532, *P* < 0.05).

However, in the ROC analysis of the predictive performance of ANI for hemodynamic adverse events, the AUC was at a low level of 0.527 (95% CI: 0.522–0.532, *P* < 0.05, Figure 3), indicating that ANI had a poor predictive performance for the occurrence of hemodynamic adverse events.

### 3.5 Correlation among ANI, SBP, and HR at different time points

Table 7 indicates the correlation coefficients among ANI, SBP, and HR. Although HR was significantly correlated with ANI (negative) and SBP (positive) at the first four time points, the correlation coefficients were all smaller than 0.30, suggesting a low correlation.

**Table 7:** Pearson’s correlation coefficients among ANI, SBP, and HR at different time points

Pearson’s correlation coefficient		ANI	SBP
Skin incision	SBP	−0.03	
	HR	−0.14*	0.24*
First trocar insertion	SBP	−0.11	
	HR	−0.25*	0.13*
Second trocar insertion	SBP	−0.01	
	HR	−0.13*	0.22*
The last trocar insertion	SBP	−0.01	
	HR	−0.16*	0.15*
5 min after the last trocar insertion	SBP	−0.03	
	HR	−0.07	0.07

*, *P* < 0.05.

## 4 Discussion

At present, minimally invasive laparoscopic surgery has become the mainstream surgical method, and the surgical procedure has been standardized. The position of the first trocar insertion is generally by making a 1 cm incision at the umbilicus, into which a 12 mm trocar was inserted. Palomba et al. have reported that in the gynecological laparoscopic surgery, there was no significant difference in hemodynamic changes in all patients at the first umbilical insertion of trocar, indicating that when the position and the size of trocar insertion are identical, the intensity of nociceptive stimuli is generally the same [30]. Camanni et al. have also found that the position and size of trocar insertion can affect the hemodynamic changes [31], suggesting that laparoscopic trocar insertion could be used as a standardized pain stimulus. In this trial, we recruited patients receiving laparoscopic surgery with at least three trocar insertions. Single-port laparoscopic surgery was excluded because the conscious perception of pain is sometimes delayed [32], making it difficult to accurately evaluate the pain of the insertion of a single trocar. There was no significant difference in the number of trocar insertions among the five groups in this study, eliminating the impact of the number of trocar insertions.

Remifentanil is an opioid drug acting on mu-type receptors, characterized by rapid onset, short duration of action, and a dose-dependent analgesic effect [33]. Therefore, different concentrations of remifentanil are usually utilized to control the levels of pain [23,24,27].

ANI can reflect the changes in the balance between analgesia and nociceptive stimuli. Our previous study showed that ANI measurement can be used for nociceptive assessment in patients with painless abortions, and ANI value was associated with the incidence of intraoperative movement [34]. In this study, under the premise of the identical intensity of nociceptive stimuli, different TCI concentrations of remifentanil were used to control the levels of pain. The concentration of remifentanil was selected based on a linear scale (1–5 ng/mL), instead of the commonly used logarithmic scale, which was based on the remifentanil TCI for the parameter set of Minto pharmacokinetic [27]. TCI method is developed based on the theoretical pharmacokinetic and pharmacodynamic to control the depth of anesthesia. Our results showed that ANI was significantly different among the five groups at the four time points (except for skin incision). In addition, ANI exhibited more linear trends (at all five time points) among the five groups (from 1 to 5 ng/mL) as compared with SBP and HR. ANI was negatively correlated with SBP or HR. The AUC of ANI was positively correlated with that of SBP and HR, respectively. Although HR significantly correlated with ANI (negative) and SBP (positive) at the first four time points, the correlation coefficients were all smaller than 0.30 (low correlation). These results suggested that under the total intravenous anesthesia after endotracheal intubation, the ANI can well reflect the intensity of nociceptive stimuli during laparoscopic trocar insertion, and could be used to assess transient pain. This also shows that under the premise that the intensity of pain stimulation is the same, different concentrations of target control remifentanil (1–5 ng/mL) are selected to form a difference in pain grade, and the change value of ANI can accurately and quickly reflect this grade difference. This finding is consistent with Boselli et al.’s findings that ANI is significantly associated with pain intensity and is a simple non-invasive method for pain assessment [17]. Our findings also implied that ANI is a better indicator of intraoperative nociceptive stimuli as compared with SBP and HR, which is consistent with Jeanne et al.’s study [19]. However, Ledowski et al. have reported that ANI, SBP, and HR have consistent sensitivity to the stimuli of skin incision [21]. This discrepancy may be attributed to the different anesthetics used in these studies. Propofol–remifentanil total intravenous anesthesia was used both in our and Jeanne et al.’s studies [19], while fentanyl and sevoflurane were used to maintain anesthesia in Ledowski et al.’s study [21]. Total intravenous anesthesia and sevoflurane-maintained anesthesia induce different stimuli to the autonomic nervous system [35], which may lead to different observations. In this study, we found that SBP changed with trocar insertion rather than with skin incision. This phenomenon may be attributed to the fact that pain response is a systemic process, and there may be a time lag between the pain and body’s corresponding response, resulting in insignificant changes in SBP when skin is incised. However, the exact mechanism remains to be further investigated. SBP measurement is invasive and is not routinely monitored in most of the operations. SBP measurement is suitable for the intraoperative and postoperative monitoring in patients with shock, severe disease, severe peripheral vasoconstriction, major surgery, or life-threatening surgery. Therefore, noninvasive ANI measurement is superior to invasive SBP measurement to monitor perioperative pain.

ANI is based on HRV. To rule out the factors that might interfere with HR, patients whose HR might be affected were excluded. In addition, during this study, enrolled patients should avoid drugs affecting HR, such as atropine and dopamine. Furthermore, the measurement of ANI was stopped at 5 min after the last trocar insertion, and surgical traction was not involved. As for the predictive performance of ANI, Jeanne et al. have demonstrated that ANI changes can predict the occurrence of hemodynamic events [36]. The contradicting results by Ledowski et al. show that ANI responds well to different levels of nociceptive stimuli, but cannot predict hemodynamic responses [21]. Our multivariate logistic regression showed that the ANI at 5 min after the last trocar insertion was the predictive factor associated with intraoperative hemodynamic adverse events (OR: 1.03, 95% CI: 1.00–1.06; *P* = 0.045). Nevertheless, ROC analysis showed that the AUC of the predictive performance of ANI for hemodynamic adverse events was only 0.527 (95% CI: 0.522–0.532, *P* < 0.05). This finding indicated that ANI responded well to transient pain under general anesthesia, but cannot be used as an independent indicator to predict the occurrence of nociceptive stimuli.

There are still some limitations to this study. First, we measured the ANI, SBP, and HR data at the very moment of the introduction of trocar, but did not measure 30–60 s after trocar introduction. In addition, due to various accidents during the research, the data of 15 cases could not be included in the final statistical analysis, which should be considered in the sample size estimation in the future study.

In summary, these results suggest that under general anesthesia, the change in ANI was consistent with changes in the balance between analgesia and nociceptive stimuli. The ANI can reflect the intensity of transient pain of laparoscopic trocar insertion. However, the ANI had poor predictive performance for the occurrence of intraoperative pain. ANI monitoring is a simple and non-invasive method for assessing intensity of transient pain, which can help anesthesiologists to improve perioperative pain management.

## Abbreviations

ANI: analgesia nociception index
ASA: American Society of Anesthesiologists
AUC: area under the curve
BIS: bispectral
HR: heart rate
HRV: heart rate variability
SBP: systolic blood pressure
TCI: target-controlled infusion

## Appendix

**Table A1:** The differences of ANI between male and female patients in each dosage group over time

ANI index		1 ng/ml		2 ng/ml		3 ng/ml		4 ng/ml		5 ng/ml	
Timing	Gender	Mean ± SD	*P*	Mean ± SD	*P*	Mean ± SD	*P*	Mean ± SD	*P*	Mean ± SD	*P*
Skin incision	Male	69.71 ± 19.72	0.565	72.63 ± 21.84	0.898	75.81 ± 18.58	0.762	66.00 ± 23.80	0.362	67.86 ± 16.90	0.359
	Female	66.33 ± 19.37		71.86 ± 20.63		74.39 ± 15.07		72.55 ± 20.77		73.03 ± 23.37	
First trocar insertion	Male	49.94 ± 17.17	0.764	52.00 ± 15.18	0.582	62.48 ± 20.69	0.387	58.17 ± 22.47	0.076	60.27 ± 20.49	0.009
	Female	51.76 ± 21.44		54.94 ± 20.27		67.52 ± 20.25		70.11 ± 19.02		75.77 ± 20.44	
Second trocar insertion	Male	48.35 ± 17.69	0.430	50.53 ± 15.97	0.550	58.19 ± 19.68	0.684	50.25 ± 12.46	0.035	55.77 ± 13.93	0.088
	Female	52.70 ± 18.59		53.49 ± 17.92		60.48 ± 19.88		61.66 ± 16.81		63.93 ± 18.43	
The last trocar insertion	Male	46.06 ± 15.06	0.271	52.78 ± 22.67	0.757	57.57 ± 14.11	0.566	49.50 ± 14.25	0.045	54.71 ± 18.83	0.131
	Female	51.27 ± 15.99		54.66 ± 19.83		60.19 ± 17.21		60.79 ± 17.23		63.67 ± 21.58	
5 mins after the last trocar insertion	Male	55.71 ± 18.39	0.341	69.79 ± 20.43	0.645	69.33 ± 16.19	0.312	65.50 ± 14.44	0.466	61.64 ± 18.48	0.051
	Female	61.48 ± 20.91		67.31 ± 17.81		74.26 ± 17.59		70.11 ± 20.04		72.80 ± 20.90	
